# Lactational High-Fat Diet Exposure Programs Metabolic Inflammation and Bone Marrow Adiposity in Male Offspring

**DOI:** 10.3390/nu11061393

**Published:** 2019-06-21

**Authors:** Hannah Hafner, Eric Chang, Zach Carlson, Allen Zhu, Mita Varghese, Jeremy Clemente, Simin Abrishami, Devika P. Bagchi, Ormond A. MacDougald, Kanakadurga Singer, Brigid Gregg

**Affiliations:** 1 Division of Diabetes, Endocrinology and Metabolism, Department of Pediatrics, University of Michigan Medicine, Ann Arbor, MI 48105, USA; hhafner@med.umich.edu (H.H.); changer@med.umich.edu (E.C.); zcarlson@med.umich.edu (Z.C.); zhual@med.umich.edu (A.Z.); mvarghes@med.umich.edu (M.V.); jerclem@med.umich.edu (J.C.); abrisham@med.umich.edu (S.A.); ksinger@med.umich.edu (K.S.); 2 Department of Molecular & Integrative Physiology, University of Michigan Medical School, Ann Arbor, MI 48105, USA; dpbagchi@med.umich.edu (D.P.B.); macdouga@med.umich.edu (O.A.M.)

**Keywords:** lactation, developmental programming, metabolism, adipose tissue, inflammation

## Abstract

Overnutrition during critical windows of development plays a significant role in life-long metabolic disease risk. Early exposure to excessive nutrition may result in altered programming leading to increased susceptibility to obesity, inflammation, and metabolic complications. This study investigated the programming effects of high-fat diet (HFD) exposure during the lactation period on offspring adiposity and inflammation. Female C57Bl/6J dams were fed a normal diet or a 60% HFD during lactation. Offspring were weaned onto a normal diet until 12 weeks of age when half were re-challenged with HFD for 12 weeks. Metabolic testing was performed throughout adulthood. At 24 weeks, adipose depots were isolated and evaluated for macrophage profiling and inflammatory gene expression. Males exposed to HFD during lactation had insulin resistance and glucose intolerance as adults. After re-introduction to HFD, males had increased weight gain and worsened insulin resistance and hyperglycemia. There was increased infiltration of pro-inflammatory CD11c^+^ adipose tissue macrophages, and bone marrow was primed to produce granulocytes and macrophages. Bone density was lower due to enhanced marrow adiposity. This study demonstrates that maternal HFD exposure during the lactational window programs offspring adiposity, inflammation, and impaired glucose homeostasis.

## 1. Introduction

Stressors during critical developmental windows are important contributors to lifetime metabolic disease risk [1]. With the increased prevalence of type 2 diabetes, understanding the contribution of early life stressors to lifetime metabolic disease risk may provide avenues for primary prevention. While in utero developmental programming events have been studied in detail, the early postnatal critical window is less well studied. The lactation period is an important time of development and reorganization of metabolic tissues [2,3,4]. This period can have an independent but equally important impact on metabolic programming [5].

Maternal metabolic disease during the reproductive cycle is becoming increasingly common with obesity impacting close to 25% of pregnancies in the United States [6]. Previous studies of high-fat diet exposure confined to the lactation period have demonstrated an increase in offspring weight gain and adiposity [7,8,9]. Neonatal overfeeding by creating small litters or giving a high-fat diet (HFD) after a gestational nutrient deficiency also show similar phenotypes with increased adiposity [10,11].

In addition to being a critical window for adipose tissue development in rodents, the early postnatal period is also a critical period of development of the neonatal immune system in both rodents and humans [12]. During this window, the possibility exists for developmental programming of adipose tissue inflammation. Studies on in utero exposure to HFD have shown an increase in fetal adipose tissue markers of inflammation [13]. Combined gestational and lactational exposure experiments have shown an increase in circulating inflammatory markers [14,15], but only a few have demonstrated inflammatory leukocytes in fat depots [16,17]. However, a detailed understanding of these adipose tissue changes with investigations into adipose tissue macrophage phenotypes with and without HFD re-challenge and fat depot differences have not been reported.

Developmental programming paradigms often include a “second-hit” which involves the introduction of a stressor after the initial programming event in order to determine if latent programmed phenotypes exist that will become apparent under situations of metabolic stress. An HFD trigger is a clinically relevant stressor for humans who have encountered developmental programming stressors during development, given the societal exposure to obesogenic diets. While some studies have described adult phenotypes in lactational high-fat diet models, detailed immune phenotyping of the adipose tissue after this “second hit” has not been reported [17,18,19].

We established a model of lactational programming with maternal HFD to understand the long-term impact of this stressor on adipose tissue morphology across several depots and the propensity toward inflammation. We also examine sex-specific differences in these metabolic programming events and the impact of a “second hit” of HFD in adult life. With these experiments, we sought to understand the long-term impact of a calorie dense, nutrient-poor diet on offspring metabolic outcomes.

## 2. Materials and Methods

### 2.1. Animal Husbandry

**Scheme 1:**
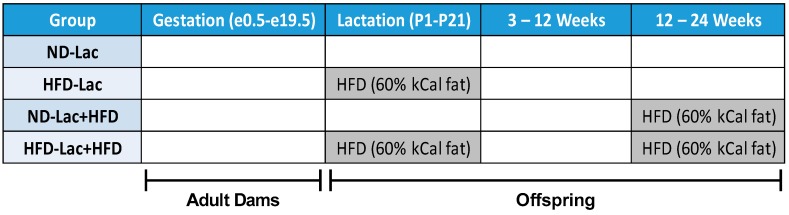
Experimental design.

Virgin C57Bl6/J mice were purchased from Jackson Laboratories (Bar Harbor, ME, USA) [3] at nine weeks of age and allowed to acclimate to the facility for at least two weeks prior to mating. Animals were kept in a ventilated rack in a facility with a 12-h light/dark cycles and were allowed free access to food and water. All procedures were performed with the approval of the University of Michigan Institutional Animal Care and Use Committee. After parturition, half of the dams were switched to a 60% HFD (D12492 Research Diets New Brunswick, NJ, USA). Control dams were maintained on 5001 (Normal Diet, ND)(13.5% kCal from fat, LabDiet, St. Loius, MO, USA). At postnatal day 21 pups were weaned onto ND and kept on this diet until age 12 weeks. At that point, a subset of the offspring was changed to 60% HFD while the rest remained on ND for an additional 12 weeks until sacrifice (Scheme 1). Just prior to necropsy, male body composition was analyzed using a Bruker Minispec LF 90II.

### 2.2. Intraperitoneal Glucose and Insulin Tolerance Tests (IPGTT, ITT)

Glucose tolerance tests were performed on offspring at approximately 8 and 22 weeks of age. Mice were fasted in the morning for 6 h prior to dextrose delivery via intraperitoneal injection at a dose of 2 g/kg for mice fed ND, and 0.66 g/kg for mice fed HFD, given excessive hyperglycemia on the standard dose. Blood glucose levels were obtained at fasting, 30, 60 and 120 min after injection from the tail vein. Insulin tolerance tests were performed at approximately 10 and 20 weeks of age. The mice were fasted for 6 h after which they received an injection of insulin lispro at a dose of 0.75 unit/kg for mice fed ND, and 1 unit/kg for mice fed HFD, given insulin resistance as previously demonstrated [20,21]. Blood glucose values were measured at fasting, 15, 30, 60 and 90 min after injection.

### 2.3. Homeostatic Model Assessment of Insulin Resistance (HOMA-IR)

Serum was collected after a 6 h fast at 8 and 23 weeks of age and analyzed for insulin concentration using a mouse ultrasensitive insulin ELISA kit (ALPCO, Salem, NH, USA) [22]. The resulting insulin level was used in conjunction with fasting glucose to calculate the homeostatic model assessment of insulin resistance using the following equation: HOMA-IR = (Insulin (uIU/mL) × Glucose (mg/dL))/405. The coefficient of variation between ELISA assays was comparable.

### 2.4. Immunofluorescence Staining

Adipose tissue explants were fixed in 1% paraformaldehyde solution for 24 h and then transferred to PBS for storage before they were used for immunofluorescence as previously described [23]. Prior to staining samples were treated with blocking solution (0.3% Triton, 5% BSA, PBS). Tissues were then stained with Caveolin-1 (BD Pharmingen, San Jose, CA, USA) [12] and Mac2 (Galectin-3 monoclonal antibody, eBioM3/38, eBioscience, San Diego, CA, USA) [24].

### 2.5. RNA Extraction, Reverse Transcription, Quantitative Real-Time PCR

RNA extraction was performed with an RNeasy lipid tissue mini kit (Qiagen, Hilden, Germany). Frozen adipose tissue was cut into 50–100 milligram pieces for RNA isolation. After RNA extraction and purification, reverse transcription was performed (Applied Biosystems, Foster City, CA, USA) to prepare cDNA and then quantitative real-time PCR analysis using glyceraldehyde-3-phosphate dehydrogenase to normalize (SYBR Green, ABI Prism 7200 Sequence Detection System, Applied Biosystems). RT-PCR primers used are detailed in Table 1.

### 2.6. Adipose Tissue Stromal Vascular Fraction (SVF) Isolation and Flow Cytometry

Adipose tissue was digested in RPMI with 1 mg/mL type II collagenase (Sigma-Aldrich, St. Louis, MO, USA) as previously described [25] on a rocking platform shaker for up to 25 min at 37 °C. The stromal vascular fraction (SVF) was separated from adipocytes by centrifugation. Following Fc blocking samples were stained for flow cytometry with anti-mouse CD45 eFluor 450 (30-F11, eBioscience) [26], anti-mouse CD11c eFluor 780 (N418, eBioscience) [27], anti-mouse CD64 PE (X54-5/7.1, BD Pharmingen) [28]. Analysis was performed using a BD Biosciences FACSAria in the Flow Cytometry Core and analyzed using FlowJo v.10 (Treestar) software [29].

### 2.7. Methocellulose Colony Forming Unit Assay

Bone marrow was flushed with Iscove’s modified Dulbecco’s medium, resuspended in MethoCult (Stem Cell technologies, Vancouver, Canada) medium for granulocyte/macrophage assay and plated with a cell density of 10,000 cells/plate. Colonies were counted seven days after plating.

### 2.8. Bone µCT

Harvested tibiae were fixed in 10% neutral-buffered formalin for 24 h, washed with water, placed in Sorensen’s phosphate buffer, pH 7.4, and stored at 4 °C prior to µCT analysis. After placement in a 19-mm diameter tube, the length of each bone was scanned using a µCT system (µCT100 Scanco Medical, Bassersdorf, Switzerland) [30]. Scan settings were as follows: voxel size 12 µm, 70 kVp, 114 µA, 0.5 mm AL filter, integration time 500 ms. Density measurements were calibrated to the manufacturer’s hydroxyapatite phantom. Analysis was performed with the manufacturer’s evaluation software, using a threshold of 180 for trabecular bone and 280 for cortical bone.

### 2.9. Bone Histology

Harvested tibiae were fixed in 10% neutral-buffered formalin for 24 h, washed with water and decalcified in 14% EDTA, pH 7.4 for 14 days. Paraffin-embedded tibiae were then sectioned at 5 m thickness, processed and stained with hematoxylin and eosin, as previously described [31].

### 2.10. Statistical Analysis

All data is shown as the mean ± standard error of the mean. A two-way ANOVA was performed initially to check for significant differences between lactational diet, HFD re-challenge, and any interactions between these exposures. Given no significant interaction, multiple comparisons were made using the Benjamini–Hochberg analysis with a false discovery rate set to 0.1. If there was a significant interaction between lactational diet and HFD re-challenge, groups were tested for normality and equal variance using the Shapiro–Wilk and Brown–Forsythe tests, respectively. Depending on these results, data were log transformed, if necessary, to produce normality and analyzed using an unpaired *t*-test, Welch’s test, or Mann–Whitney U-test. Repeated measures of weight and timed metabolic tests were analyzed using a two-way ANOVA. Significance was determined as *p* < 0.05. All statistical analysis was performed using GraphPad Prism 7.00 software [32].

## 3. Results

### 3.1. Male Lactation HFD Offspring Show Insulin Resistance and Mild Glucose Intolerance on Normal Diet

**Figure 1:**
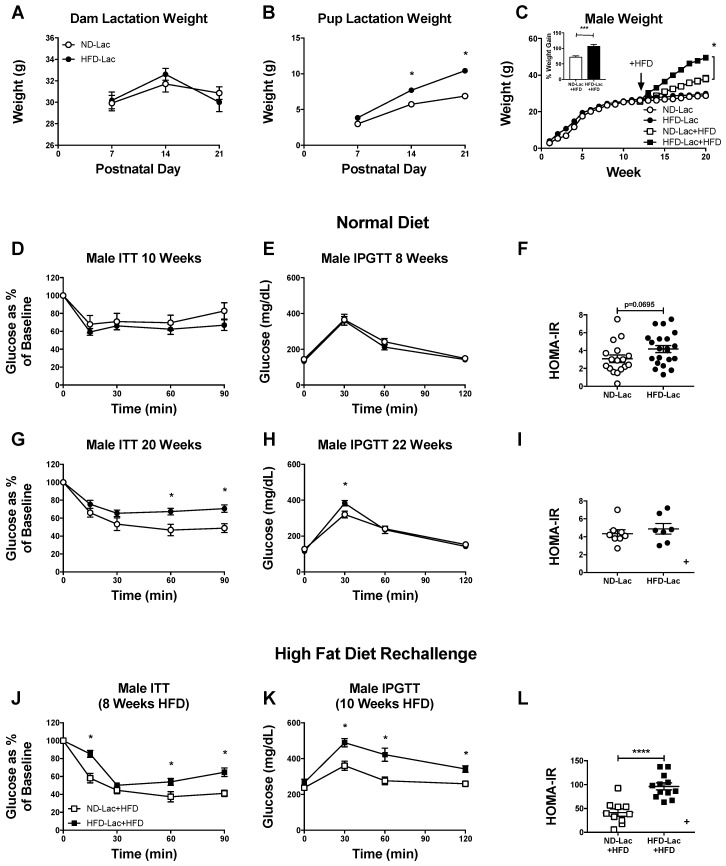
Male lactation high-fat diet (HFD) offspring show insulin resistance and mild glucose intolerance on a normal diet. Analyzed experimental groups: ND-Lac (white circles), HFD-Lac (black circles), ND-Lac+HFD (white squares), HFD-Lac+HFD (black squares). Weekly weights of dams during the lactation period starting on postnatal day 7 (**A**). Weekly weights of male and female offspring during the lactation period (**B**). Weekly weights of male offspring throughout adulthood, arrow indicates the start of HFD re-challenge (**C**). Data collected from males maintained on a normal diet (**D**–**I**). Insulin tolerance tests (ITT) at 10 weeks of age, 6 h fast, 0.75 U/kg insulin, ND-Lac *n* = 7, HFD-Lac *n* = 10 (**D**). Intraperitoneal glucose tolerance tests (IPGTT) at eight weeks of age, 6 h fast, 2 g/kg dextrose, ND-Lac *n* = 8, HFD-Lac n = 13 (**E**). HOMA-IR at eight weeks of age measured after 6 h fast (**F**). ITT at 20 weeks of age, ND-Lac *n* = 5, HFD-Lac *n* = 12 (**G**). IPGTT at 22 weeks of age, ND-Lac *n* = 4, HFD-Lac *n* = 7 (**H**). HOMA-IR at 23 weeks of age (**I**). Data collected from males re-challenged with HFD at 12 weeks of age (+HFD) (**J**–**L**). ITT after eight weeks of HFD feeding, 1 U/kg, ND-Lac+HFD *n* = 10, HFD-Lac+HFD *n* = 11 (**J**). IPGTT after 10 weeks of HFD feeding, 0.66 g/kg dextrose, ND-Lac+HFD *n* = 8, HFD-Lac+HFD *n* = 6 (**K**). HOMA-IR after 11 weeks of HFD feeding (**L**). * *p* < 0.05, *** *p* < 0.001, **** *p* < 0.0001. + indicates a significant interaction between lactation exposure and HFD re-challenge.

Dams received HFD for 21 days during the lactation period. This exposure did not alter maternal body weight (Figure 1A), but the growth of male and female offspring was increased in the HFD-Lac group (Figure 1B). Upon weaning onto a normal diet, the weights of male offspring converged after weaning and continued to have no differences through the end of the experiment at six months of age (Figure 1C). Despite having no difference in body weight, insulin sensitivity or glucose tolerance in early adulthood (Figure 1D,E), the male HFD-Lac offspring tended to have increased HOMA-IR at eight weeks of age (Figure 1F). When we continued to follow the male offspring on a normal diet, we found that HFD-Lac males developed insulin resistance by ITT at 20 weeks (Figure 1G) with mild glucose intolerance at 22 weeks (Figure 1H). Interestingly, the increase in HOMA-IR observed earlier in life was not apparent at 23 weeks of age (Figure 1I). When the male mice were put on a high-fat diet re-challenge (+HFD) at 12 weeks of age, the metabolic phenotypes observed in males on a normal diet were exacerbated. Re-challenged HFD-Lac males were significantly heavier than re-challenged ND-Lac males (Figure 1C) with insulin resistance by ITT after eight weeks of HFD (Figure 1J) and severe glucose intolerance by IPGTT at 10 weeks of HFD (Figure 1K) with a significantly elevated HOMA-IR (Figure 1L). Re-challenged HFD-Lac males had increased liver alanine aminotransferase (ALT), 35.6 ± 7.068 U/L, compared to ND-Lac, 13.8 ± 2.956 U/L. There was also no difference in aspartate transaminase (AST) (ND-Lac 92.6 ± 13.35 U/L vs. HFD-Lac 141.2 ± 22.47 U/L) or circulating triglyceride levels (ND-Lac 93.6 ± 7.434 U/L vs. HFD-Lac 100.2 ± 7.971 U/L) in re-challenged mice. Overall, maternal high-fat diet during the lactation period appears to predispose male offspring to develop insulin resistance later in life while on a normal diet which is exacerbated by a second exposure to HFD. This is likely a programmed effect on peripheral insulin sensitivity given that in vivo insulin secretion was elevated in HFD-Lac males regardless of diet and no difference was detected in pancreatic beta-cell mass in HFD re-challenge males (ND-Lac 2.54 ± 0.25 mg vs. HFD-Lac 2.77 ± 0.24 mg).

### 3.2. Female Offspring Had no Evident Metabolic Changes after Lactational HFD Exposure

**Figure 2:**
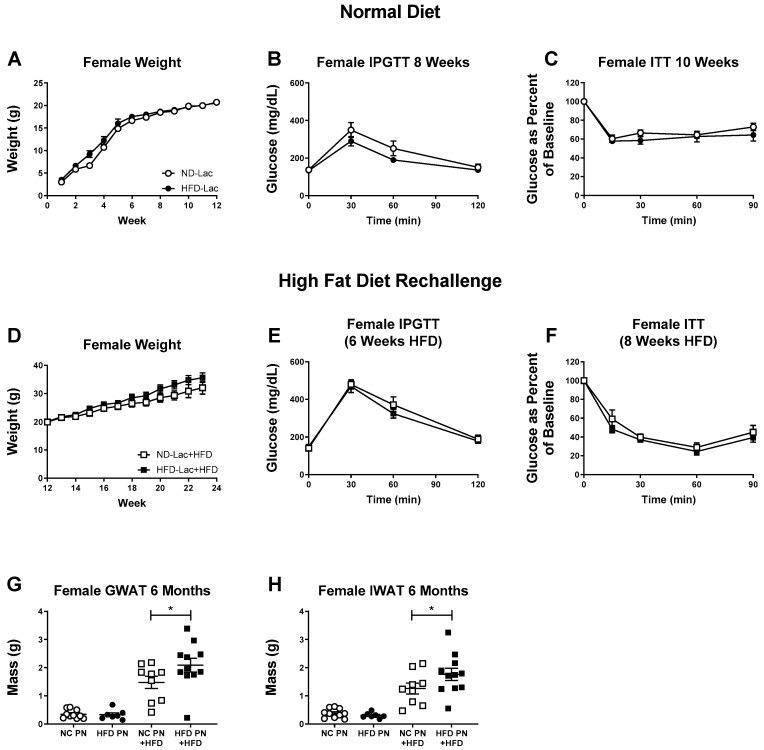
Female offspring had no evident metabolic programming after lactational HFD exposure. Analyzed experimental groups: ND-Lac (white circles), HFD-Lac (black circles), ND-Lac+HFD (white squares), HFD-Lac+HFD (black squares). Data collected from females maintained on a normal diet (**A**–**C**). Weekly weights (**A**). IPGTT at eight weeks, 6 h fast, 2 g/kg dextrose, ND-Lac *n* = 14, HFD-Lac *n* = 16 (**B**). ITT at 10 weeks, 6 h fast, 0.75 U/kg insulin, ND-Lac *n* = 10, HFD-Lac *n* = 9 (**C**). Data collected from females re-challenged with HFD at 12 weeks of age (+HFD) (**D**–**F**). Weekly weights starting at the time of HFD feeding (**D**). IPGTT after six weeks of HFD feeding, 2 g/kg dextrose, ND-Lac+HFD *n* = 9, HFD-Lac+HFD *n* = 11 (**E**). ITT after eight weeks of HFD feeding, 1 U/kg insulin, ND-Lac+HFD *n* = 9, HFD-Lac+HFD *n* = 11 (**F**). Gonadal (**G**) or inguinal (**H**) white adipose depot weights collected at six months of age or after 12 weeks of HFD feeding. * *p* < 0.05.

Female HFD-Lac offspring were heavier during the suckling period, and their weights converged upon weaning, similar to males in the same group (Figure 2A). Female offspring maintained on a normal diet had no significant changes in glucose tolerance by IPGTT and no differences in insulin sensitivity as measured by ITT (Figure 2B,C). When placed on HFD re-challenge the HFD-Lac offspring tended to have increased body weight, but this did not reach statistical significance (Figure 2D). They also had no differences in glucose tolerance or insulin sensitivity after HFD feeding (Figure 2E,F). At necropsy, there were no significant differences in the weights of gonadal or inguinal white adipose tissue depots between groups on a normal diet (Figure 2G). Re-challenged HFD-Lac females, however, had increased weights in both of these depots (Figure 2H). Maternal high-fat diet differentially programs insulin resistance between males and females. Females exposed to HFD early in life do not display exacerbated insulin resistance as do male offspring, however, they are more susceptible to increased adiposity when re-exposed to HFD later in life.

### 3.3. Re-Challenged Male HFD-Lac Offspring Have Increased Fat Mass

**Figure 3:**
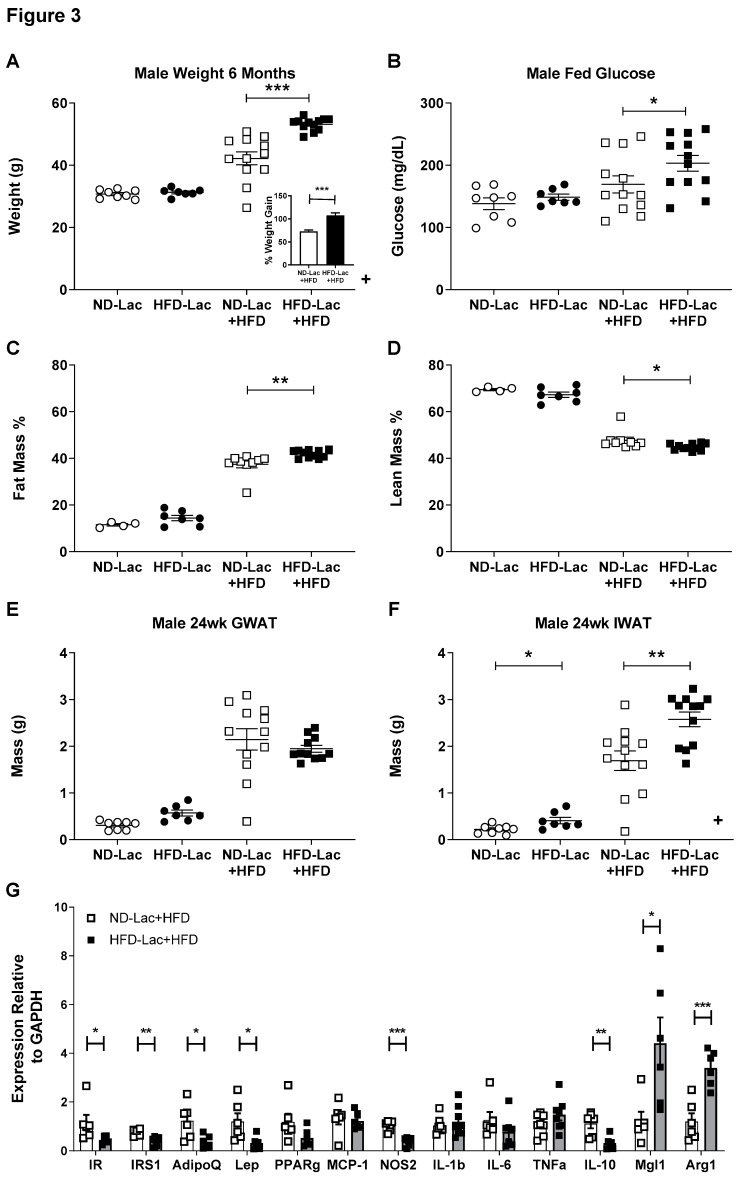
Male Lac-HFD offspring have increased fat mass. Data collected from males on either a normal diet or high-fat diet (+HFD) at six months of age. Weight, inset-percent weight gain over the course of HFD re-challenge (**A**). Fed glucose (**B**). Fat mass percentage (**C**). Lean mass percentage (**D**). Gonadal white adipose tissue (GWAT) weight (**E**). Inguinal white adipose tissue (IWAT) weight (**F**). qPCR on GWAT from males fed HFD (**G**). * *p* < 0.05, ** *p* < 0.01, *** *p* < 0.001. + indicates a significant interaction between lactation exposure and HFD re-challenge.

All male offspring on either a normal diet or high-fat diet were sacrificed at six months of age. At that point, the HFD-Lac males that were fed HFD were significantly heavier than ND-Lac males fed HFD (Figure 3A). Over the course of the HFD re-challenge, HFD-Lac males gained a higher percentage of their original body weight as compared to ND-Lac males (Figure 3A inset). HFD re-challenge led to an increase in fed glucose levels in HFD-Lac males (Figure 3B). Body composition analysis of male offspring revealed that fat mass and lean mass were only affected after HFD re-challenge (Figure 3C,D). HFD-Lac males had increased fat percentage and decreased lean mass percentage after 12 weeks of HFD feeding (Figure 3C,D). Gonadal and inguinal white adipose depots were next examined. The gonadal depot was not different between lactation exposure groups fed either diet (Figure 3E). The inguinal depot was heavier in HFD-Lac males on a normal diet and remained significantly heavier after 12 weeks of HFD re-challenge (Figure 3F). These results suggest that male offspring exposed to HFD during the lactation period are more susceptible to diet-induced obesity and increased adiposity.

Considering the alterations in glucose homeostasis, insulin sensitivity, and body composition, we next analyzed RNA from the GWAT of males on a high-fat diet by qPCR for a number of genes related to metabolism and inflammation (Figure 3G). Levels of insulin receptor (*Insr*), insulin receptor substrate 1 (*Irs1*), and adiponectin (*AdipoQ*) mRNA were significantly reduced by exposure to HFD during lactation. Interestingly, HFD-Lac males on a high-fat diet also showed reduced expression of leptin (*Lep*) in the GWAT (Figure 3G). It is possible that leptin is differentially expressed in other adipose tissue depots such as IWAT, especially considering the increase in weight after HFD feeding in both groups. Surprisingly, circulating leptin levels were no different amongst any of the HFD re-challenged animals (ND-Lac 31.64 ± 1.33 ng/mL vs. HFD-Lac 30.75 ± 1.03 ng/mL). We also observed decreased expression of the inflammatory marker *Nos2*, but no change in the expression of *MCP-1*, *IL-6*, *IL-1b*, or *TNFα*. Typical markers of resident, anti-inflammatory macrophages *Mgl1* showed a decreased expression, while expression of *Arg1* and *Il10* were increased in HFD-Lac males (Figure 3G). This may also reflect a dampening in pro-inflammatory cytokine production by pro-inflammatory cells as can be seen in the setting of weight loss [33]. Reduction in expression of *Insr*, *Irs1,* and *AdipoQ* may possibly contribute to the severe insulin resistance that developed after a second exposure to HFD. The profile of inflammatory and anti-inflammatory signals may point to a shift in macrophage polarization in males exposed to a maternal HFD.

### 3.4. Enhanced Myeloid Inflammation in HFD-Lac Males Challenged to HFD

**Figure 4:**
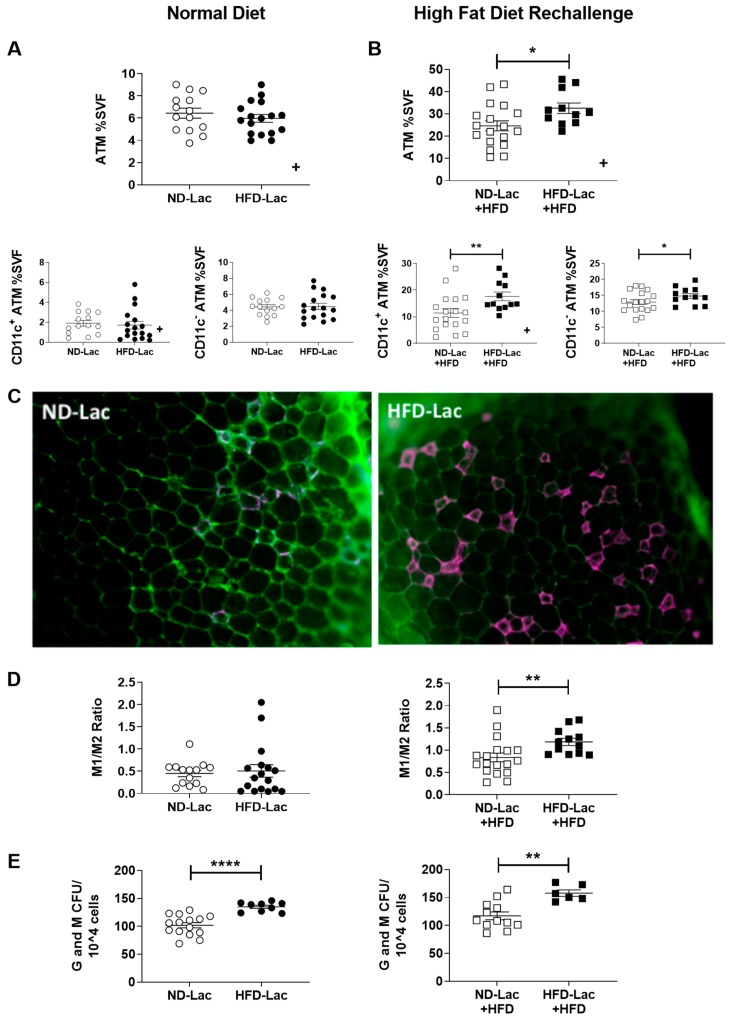
Enhanced myeloid inflammation in Lac-HFD males challenged to HFD. Data collected from six-month-old males on either a normal diet or high-fat diet (+HFD). Total adipose tissue macrophages (ATMs), CD11c^+^ ATMs and CC11c^−^ ATMs in GWAT of males fed a normal diet (**A**) or fed HFD (**B**). Representative immunofluorescent images of crown-like structure formation in GWAT (Green Caveolin, Magenta Mac2) (**C**). Ratio of pro-inflammatory M1 to anti-inflammatory M2 macrophages in GWAT (**D**). Granulocyte and monocyte colony forming units from bone marrow (**E**). * *p* < 0.05, ** *p* < 0.01, **** *p* < 0.0001. + indicates a significant interaction between lactation exposure and HFD re-challenge within the analyzed endpoint.

To next understand the impact on macrophage phenotype we evaluated macrophages in visceral GWAT pads from both normal diet and HFD challenged groups. In normal diet groups, total ATMs and ATM subtypes were similar in ND-Lac and HFD-Lac groups (Figure 4A). When re-challenged to HFD in adulthood HFD-Lac mice had increased total ATMs as well as increased CD11c^+^ pro-inflammatory and, to a lesser extent, increased CD11c^−^ anti-inflammatory ATMs (Figure 4B). The CD11c^+^ subtype of ATMs is known to have enhanced inflammatory cytokine production and a propensity to form crown-like structures (CLS) in adipose tissue as seen in IF images (Figure 4C). Another common way to consider the pro-inflammatory (M1) to anti-inflammatory (M2) make-up is to evaluate the ratio of these macrophage types. While there was an increase in both ATM subtypes, the ratio of M1:M2 macrophages was higher in HFD-Lac mice on HFD (Figure 4D). Given that these CD11c^+^ ATMs are typically recruited from the bone marrow, we next evaluated bone marrow preference for myelopoiesis using in vitro methocellulose colony forming assays. These studies demonstrated, as expected, that the HFD-Lac mice had enhanced myeloid colonies consistent with the increased inflammatory tone in the adipose tissue after HFD re-challenge (Figure 4E). Exposure to a maternal HFD during lactation seems to program the bone marrow of offspring for enhanced myelopoiesis regardless of diet in adulthood. This may be linked to increased macrophage infiltration into adipose depots when re-exposed to HFD, leading to the observed increase in both pro- and anti-inflammatory ATMs with a strong M1 polarization.

### 3.5. HFD Re-Challenge after Exposure to HFD during Lactation Causes Long-Term Bone Loss and Expansion of Bone Marrow Adiposity

**Figure 5:**
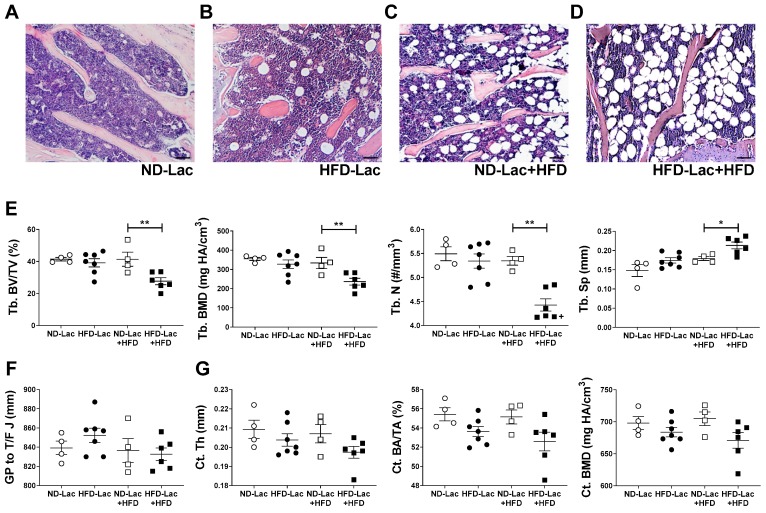
HFD re-challenge after exposure to HFD during lactation causes long-term bone loss and expansion of bone marrow adiposity. Representative sections of proximal tibiae from (**A**) ND-Lac, (**B**) HFD-Lac, (**C**) ND-Lac+HFD, and (**D**) HFD-Lac+HFD mice were stained with H&E and are shown at 100× magnification; scale bar, 100 μm. Tibial trabecular bone volume fraction (Tb. BV/TV), bone mineral density (Tb. BMD), trabecular number (Tb. N) and separation (Tb. Sp) were measured with μCT (**E**). Tibial length was approximated by the distance from the growth plate (GP) to the tibia/fibula junction (T/F J) (**F**). Mid-tibia cortical bone area (Ct. BA/TA), thickness (Ct. Th), and bone mineral density (Ct. BMD) were also measured with μCT (**G**). * *p* < 0.05, ** *p* < 0.01. + indicates a significant interaction between lactation exposure and HFD re-challenge.

Studies are increasingly identifying important functional interactions between metabolic and skeletal homeostasis. Given global changes in adiposity in the male offspring by mini-spec but no change in GWAT size, the bone marrow niche was examined to determine if this adipose depot was impacted by the lactational HFD exposure. We, therefore, examined the effects of HFD exposure during lactation on bone parameters and marrow adiposity. HFD feeding during lactation caused a mild increase in tibial regulated bone marrow adipocytes (rBMAs) (Figure 5A,B), but did not affect cortical or trabecular bone parameters. Whereas post-weaning exposure to HFD caused a robust increase in rBMAs, the expansion was more pronounced in mice that had been previously exposed to HFD during lactation (Figure 5C,D). The increase in tibial rBMAT following HFD re-challenge was accompanied by decreased trabecular bone volume fraction (Tb. BV/TV), bone mineral density (Tb. BMD), and trabecular number (Tb. N) and increased spacing between trabeculi (Tb. Sp) (Figure 5E). Tibial constitutive bone marrow adipose tissue (BMAT) was not affected by HFD exposure (data not shown). Bone length (Figure 5F) and tibial cortical thickness (Ct. Th), area (Ct. BA/TA), and bone mineral density (Ct. BMD) (Figure 5G) were not significantly different across experimental groups. Taken together, these data suggest that HFD feeding during lactation may increase susceptibility to alterations in the bone marrow niche, which is exacerbated by HFD re-exposure later in life. These changes within the bone marrow may have long-lasting implications for bone health.

## 4. Discussion

In the present study, we found that lactational HFD exposure programs male offspring to have increased adipose tissue weights and insulin resistance while on a normal diet. Visceral GWAT was unchanged in HFD-Lac animals maintained on either diet while IWAT was expanded in both control and HFD re-challenge animals. When given an HFD re-challenge, the male offspring have metabolic decompensation with severe glucose intolerance and worsened insulin resistance. These results are similar to those of other studies that have shown insulin resistance and increased body fat after a lactational HFD exposure [8,9], however the only group that reported female offspring outcomes reported a similar phenotype to males, which we did not detect in our studies [34]. Our studies demonstrated a clear sex difference between male and female offspring with females showing early growth acceleration but protection from long-term glucose homeostasis abnormalities despite having an increase in visceral fat which was not detected in the males. Future studies will be designed to understand the factors that contribute to sex differences in lactational HFD programming.

We went further to examine the extent to which meta-inflammation was altered by profiling adipose tissue inflammation in this model. Our work agrees with the findings of Monks et al. who reported increased crown-like structures in the visceral white adipose tissue after a lactational high-fat diet exposure [17]. Postnatal overfeeding by reducing litter size has also been linked with increased TNF-alpha and IL-6 in the offspring mesenteric WAT [18]. When we performed flow cytometry studies the macrophage infiltration was characterized by an increase in both CD11c^+^ pro-inflammatory and CD11c^−^ anti-inflammatory macrophages with M1 polarization. In HFD re-challenge males there was also an increase in resident M2 macrophages, as can be seen with HFD feeding [35] but M1 were predominant. We also discovered that the bone marrow from the lactational HFD exposure group is primed to produce more granulocytes and monocytes both during the normal diet and HFD re-challenge phases. This may contribute to the observed inflammation after HFD re-challenge as the bone marrow shows evidence of programming to generate more inflammatory cells. We have previously demonstrated priming of the bone marrow macrophage progenitors by high-fat diet in an adult model [36] and this priming also appears to occur as a result of dietary exposures during developmental windows. The ability of maternal HFD to program increased myelopoiesis in offspring has been shown in a fetal study after pregestational and gestational high-fat diet exposure [37] but not with an exclusively lactational exposure. Others have demonstrated using a sheep model that perirenal WAT toll-like receptor 4 (*TLR4*) expression was shown to peak at postnatal day 30 and was influenced by maternal diet during late gestation [38]. This work shows the important influence that maternal diet during lactation has on shaping offspring macrophage priming and susceptibility to adipose tissue inflammation, however the aspects of the programmed immune cell phenotype may vary depending on the developmental window of exposure.

When we performed qPCR studies on the GWAT we found that pro-inflammatory gene expression was in most cases no different or reduced in the HFD programmed males. There were however increases detected in *Arg1*, thought to be important for M2 function and protection from immune cell-mediated tissue damage, and *IL-10*, thought to be an anti-inflammatory cytokine [39]. Overall, the genes for alternative macrophage activation give a mixed picture perhaps indicating that the macrophage programming in the lactational window increases CD11c^+^ ATMs but might also enhance resident macrophage activation profiles as well. This emphasizes that current immune cell phenotyping may not be able to yield a complete picture of macrophage phenotypes from this programming window and that future studies should examine global immune activation in these animals. In addition, given that we measured gene expression in whole adipose tissue, both adipocyte and other immune cells in the stromovascular fraction may have contributed to the measured gene expression profiles. There was also no difference in *MCP-1* expression in GWAT, an important call signal for macrophage recruitment to tissues, after 12 weeks of HFD. It is possible that this level would differ between groups earlier in the HFD exposure when the macrophages are initially being recruited. When adipokine expression was measured there was a decrease in adiponectin and leptin in HFD-Lac re-challenge. In addition, when metabolic genes were measured there was a decrease in GWAT insulin receptor and *IRS-1* in HFD-Lac re-challenge. These findings agree with the insulin resistance and lack of adipocyte size changes detected in the male offspring.

Studies are increasingly identifying important functional interactions between the variety of cells within the bone and their role in local bone development and metabolism. Bone marrow adipose tissue (BMAT) is a unique adipose depot that resides within the bone cavity, alongside hematopoietic and bone cells, and contributes to local and whole-body metabolism [40,41]. At birth, bone marrow is largely comprised of hematopoietic cells, but the number of bone marrow adipocytes (BMAs) increases dramatically during postnatal development. BMAT can be defined by two subpopulations: constitutive BMAT (cBMAT) and regulated BMAT (rBMAT). Whereas cBMAT adipocytes are largely unaffected by the environment, rBMAs develop within the trabecular bone marrow throughout life and are dynamically regulated by a wide variety of nutritional, genetic, and endocrine cues [40,41].

In the present study, we examined for the first time the effects of lactational HFD exposure on bone marrow adiposity and its relation to bone health in mice. Indeed, bone compartments may be impacted by MAT expansion and increased hematopoiesis, as demonstrated by our methocellulose CFU assay. We found that maternal HFD feeding during the three-week lactation window causes dramatic rBMAT expansion and decreased trabecular bone volume and density following HFD re-challenge later in life. Importantly, mice exposed to HFD only later in life did not exhibit trabecular bone loss, despite a relative increase in rBMAT. Although these results largely align with previous rodent studies, which have shown that maternal HFD feeding during pre-conception and gestation is associated with stunted skeletal development, decreased trabecular and cortical bone mineral density, and decreased overall bone mass in offspring [42,43], our data are novel in that they demonstrate that a brief exposure to HFD early in life is sufficient to predispose offspring to poor bone health.

The mechanisms underlying increased rBMAT and decreased trabecular bone in pups exposed to HFD during lactation and later in life remain unknown. However, several plausible hypotheses can be put forward. BMAs and osteoblasts are derived from common mesenchymal progenitors. Pro-adipogenic signals stimulate adipogenesis and inhibit bone formation, whereas anti-adipogenic signals impair adipogenesis and promote osteogenic differentiation [40]. Evidence suggests that perinatal developmental programming allows offspring to make inferences about the postnatal energetic environment based on maternal cues [44], and numerous studies have shown that maternal HFD feeding during gestation induces offspring obesity [45,46,47]. It is possible that since BMAT develops postnatally, early exposure to HFD may contain signals that cause nutritional programming of progenitor cells within the bone marrow niche, committing them to a pro-adipogenic lineage. These committed progenitors may more readily develop into mature adipocytes when re-challenged with HFD later in life. Alternatively, the effects on bone may be secondary to altered whole-body metabolism or endocrine signaling via leptin, glucocorticoids or osteocalcin, for example. Leptin may be of particular interest as it can influence bone mass both directly and indirectly [48], and since maternal HFD feeding has been shown to induce leptin insensitivity in offspring [45]. Altered epigenetic regulation via DNA methylation and histone modifications should also be explored as possible mechanisms underlying long-term susceptibility to poor bone health following HFD exposure during lactation.

## 5. Conclusions

With this study, we have found that brief lactational exposure to HFD is capable of long-term programming of adiposity, adipose tissue inflammation, bone marrow responses and the individual compartments of the bone. This work highlights the importance of neonatal exposures and the capacity for milk-borne factors to act as a powerful programming agent that can impact long term metabolic risk. Further studies are warranted to understand the specific triggers of this immune priming and the capacity for rescue from this neonatal stressor.

## Figures and Tables

**Table 1:** qPCR Primer Sequences.

Gene	Forward 5′-3′	Reverse 5′-3′
*IR*	TTTGTCATGGATGGAGGCTA	CCTCATCTTGGGGTTGAACT
*IRS1*	CGATGGCTTCTCAGACGTG	CAGCCCGCTTGTTGATGTTG
*AdipoQ*	GCAGGCATCCCAGGACATC	GCGATACATATAAGCGGCTTCT
*Lep*	GAGACCCCTGTGTCGGTTC	CTGCGTGTGTGAAATGTCATTG
*PPARg*	GGAAGACCACTCGCATTCCTT	TCGCACTTTGGTATTCTTGGAG
*MCP-1*	TTAAAAACCTGGATCGGAACCAA	GCATTAGCTTCAGATTTACGGGT
*NOS-2*	CCAAGCCCTCACCTACTTCC	CTCTGAGGGCTGACACAAGG
*IL-1b*	AAATACCTGTGGCCTTGGGC	CTTGGGATCCACACTCTCCAG
*IL-6*	TAGTCCTTCCTACCCCAATTTCC	TTGGTCCTTAGCCACTCCTTC
*TNFa*	TAGCCCACGTCGTAGCAAAC	ACAAGGTACACCCCATCGGC
*Mgl1*	TGAGAAAGGCTTTAAGAACTGGG	GACCACCTGTAGTGATGTGGG
*Arg1*	CTCCAAGCCAAAGTCCTTAGAG	AGGAGCTGTCATTAGGGACATC
*IL-10*	GCTCTTACTGACTGGCATGAG	CGCAGC TCT AGG AGC ATG TG
*GAPDH*	TGAAGCAGGCATCTGAGGG	CGAAGGTGGAAGAGTGGGAG
